# Barriers and Facilitators of Quality Family-Centered Communication in Pakistan

**DOI:** 10.1200/GO.23.00178

**Published:** 2023-11-02

**Authors:** Dylan Graetz, Alia Ahmad, Muhammad Rafie Raza, Ambreen Hameed, Asma Naheed, Atoofa Najmi, Afia tul Quanita, Shabnam Munir, Gia Ferrara, Courtney Staples, Carlos Rodriguez Galindo, Syed Ahmer Hamid, Sima Jeha, Jennifer W. Mack

**Affiliations:** ^1^ St Jude Children's Research Hospital, Memphis, TN; ^2^ Children's Hospital Lahore, Lahore, Pakistan; ^3^ The Indus Hospital, Karachi, Pakistan; ^4^ Dana Farber Cancer Institute/Boston Children's Hospital, Boston, MA

## Abstract

**PURPOSE:**

Communication is a fundamental aspect of patient- and family-centered care. Unfortunately, there is a dearth of evidence regarding pediatric cancer communication in low- and middle-income countries, where over 90% of all children with childhood cancer live. The purpose of this study was to explore barriers and facilitators of quality communication within two pediatric cancer centers in Pakistan.

**METHODS:**

Semistructured interviews were conducted with 20 multidisciplinary pediatric cancer clinicians and 18 caregivers of children with cancer at Children's Hospital of Lahore and Indus Hospital in Karachi, Pakistan. Interviews were conducted in English or Urdu, audio-recorded, transcribed, and translated to English. Two researchers coded each transcript using an inductively derived codebook. Thematic content analysis focused on barriers and facilitators of high-quality communication.

**RESULTS:**

Pakistani clinicians and caregivers identified factors that affected the quality of patient-centered cancer communication. These included structural factors including setting, available interpreters, documentation, patient volume, teamwork, and financial support. Clinician-level communication barriers and facilitators included communication training, clinician distress/boundaries, and the ability to have recurrent conversations. Patient or family characteristics affecting communication included education, income status, primary language, and geography; the child's specific disease type; and relational elements such as social support, empowerment, and split decision makers. Participants identified existing or potential interventions related to each factor.

**CONCLUSION:**

Multilevel factors serve as either barriers or facilitators for pediatric cancer communication in Pakistan. Identification of these elements of communication is an essential step toward interventions aimed at improving patient- and family-centered care in resource limited settings.

## Teaser

Identified barriers, facilitators, and interventions to improve pediatric cancer communication in Pakistan.

## INTRODUCTION

High-quality communication is an essential component of family-centered care.^1^ In pediatrics, effective communication helps the health care team share knowledge, assist families with decisions, and build rapport. It has been recognized by the American Academy of Pediatrics as a skill critical to safe practice^2^ and defined as a domain of competence by the American Pediatric Association.^3^ Communication has been similarly emphasized in cancer care, where decisions are complex, evidence may be limited, and shared decision making depends on an understanding of family values.^4^ In pediatric cancer, high-quality communication reduces parental distress^5^ and increases parental trust in the medical team.^6^

**CONTEXT**

**Key Objective**
What are barriers and facilitators of family-centered communication for children with cancer in Pakistan?
**Knowledge Generated**
Using interviews with parents and clinicians of children with cancer in Pakistan, we identified multilevel barriers and facilitators to high-quality communication. Barriers and facilitators included structural factors, clinician factors, and patient and family factors, each of which may be addressed with targeted interventions.
**Relevance**
Understanding barriers and facilitators of high-quality communication in Pakistan can allow clinical teams to implement changes that address challenges and support best practice.

Although 90% of children with cancer live in low- and middle-income countries (LMICs),^7^ and it is well established that culture and context influence communication,^8,9^ there is a dearth of literature exploring family-centered communication in LMICs.^10^ In these settings, delayed diagnosis and abandonment of therapy contribute to high mortality rates.^11^ Thus, a focus on communication not only has the potential to improve quality of care but may also play a role in decreasing abandonment and improving access to care. In addition, pediatric cancer care in LMICs is often complicated by fewer treatment options; this contributes to complex decision making if the standard of care is not available and an analysis of risk and benefits that accounts for unique cultural and clinical considerations. This goal of this study was to explore barriers and facilitators of high-quality family-centered cancer communication in Pakistan, a diverse lower-middle income country in which families face significant hardships and socioeconomic challenges.^12^

## METHODS

### Setting and Participants

Pakistan is a predominately Muslim country in which >20% of the population lives below the international poverty line. The official languages include Urdu and English; however, it is estimated that >75 different languages are spoken. Pakistan has a population of approximately 200 million people, and an estimated 8,000-12,000 children develop cancer in Pakistan each year.^13^ While national outcome data are not available, the childhood cancer survival rate is estimated below 50%.^14^

This study was conducted at two pediatric cancer referral hospitals in Pakistan: Indus Hospital and Health Network in Karachi and Children's Hospital of Lahore. Each hospital cares for >1,200 newly diagnosed patients a year. Indus is a nonprofit hospital supported by donations, whereas Children's Hospital Lahore is a public hospital supported by the government. All children receive cancer care free of charge at both institutions. Participants included multidisciplinary pediatric oncology clinicians from each hospital and caregivers of children younger than 19 years with cancer at Indus who were interviewed within 8 weeks of diagnosis. Purposive sampling was used to ensure representation from a range of clinical disciplines and parents of children with a variety of diagnoses and diverse religious and socioeconomic backgrounds.

### Study Design and Data Collection

Interviews with clinicians were conducted in English over a video-conferencing platform by members of the St Jude research team who did not supervise participants. Interviews with families were conducted in Urdu by members of the research team in Pakistan. All interviewers received training in qualitative data collection. Interviews were audio-recorded, transcribed, and translated to English. Semistructured interview guides explored communication practices including cultural beliefs and local traditions and multilevel factors perceived to positively or negatively affect communication. To identify potential interventions to improve communication, clinicians were asked, “If you could change one thing or make one aspect of communication easier, what would it be?” Parents were asked, “If you had the opportunity now to speak with other parents of a child recently diagnosed with cancer, what would you tell them about talking to the doctors and nurses here? What advice would you give them?”

### Data Analysis

Three authors (D.G., C.S., and G.F.) iteratively read transcripts and inductively identified codes on the basis of recurrent themes. Codes were conceptually defined and refined through iterative review and application to 17 transcripts. Identified codes were categorized into three general categories of factors affecting quality communication: structural factors, clinician-level factors, and patient/family level factors. Two researchers (C.S. and G.F.) independently coded each transcript using the final codebook (available upon request) and MAXQDA software (VERBI, Berlin, Germany). Discrepancies were resolved by consensus and a third-party adjudicator (D.G.) as necessary. Consolidated Criteria for Reporting Qualitative Studies guidelines were followed to ensure rigor.^15^

## RESULTS

Twenty multidisciplinary clinicians and 18 caregivers were interviewed for this study. Demographics are presented in Table 1. Participants discussed multilevel factors that contributed to the quality of family-clinician communication. All determinants were described by both clinicians and caregivers and served as either barriers or facilitators of high-quality communication.

**TABLE 1:**
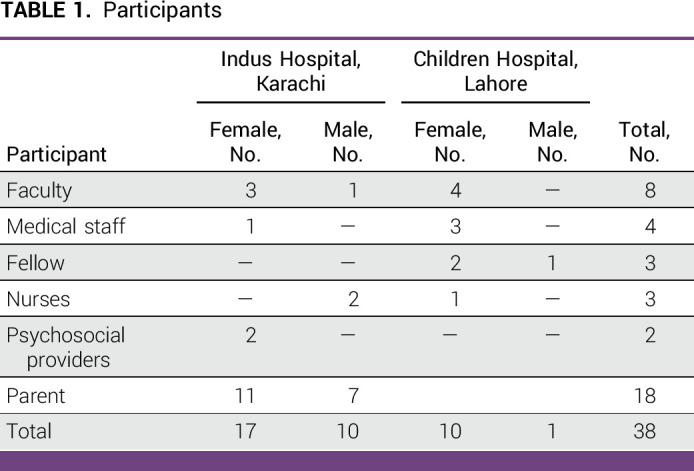
Participants

### Structural Barriers and Facilitators of Communication

Clinicians and caregivers described structural characteristics that facilitated or hindered communication. These included the physical setting for communication, availability of interpreters, documentation, patient volume or time, teamwork, and financial support.

When describing settings in which they had important conversations, clinicians and caregivers highlighted the importance of privacy and having a separate room to sit and talk. Unfortunately, because of both physical space constraints and patient volume, privacy was not always possible and many conversations occurred in busy emergency departments or on inpatient wards with multiple families present.

Language barriers between families and staff hindered communication. Neither institution had formal interpreter services; instead, staff and family members were often used as informal translators. As one father described, “When I would not understand, there was a team member in the staff who was a Pathan. He would converse in Pashto…and tell me to do this and that.”

Caregivers described taking their child's medical records from one hospital to another to facilitate information exchange and clinical care, “We showed the report from [outside hospital]…As soon as we showed that report, they admitted my daughter…and the doctor said that it is [cancer]” (mother, Indus). Clinicians used medical records to prepare for communication events: “I review that beforehand for the first interaction, and then I have that detailed information that I can talk to them in detail about the diagnosis” (oncologist, Indus). At times, a lack of documentation was cited as a barrier to communication; there was often no record of what had been covered in previous encounters.

One of the most frequently discussed barriers to quality communication at both institutions was the high patient volume and the lack of time clinicians had to spend with each family. While some caregivers described clinicians giving as much of an hour of their time to sit and talk, others noticed how hurried their health care team was.

Although multidisciplinary teams communicated with families at both institutions, Indus benefited from a psychosocial team, whereas Children's Hospital Lahore lacked this support. Most of the time, a large team was described by parents and clinicians as an advantage that offsets the limited time each clinician had to spend with a family. However, there were a few instances in which a large team was described as a challenge to communication if team members did not provide a consistent message.

Finally, a theme raised by caregivers and clinicians alike was the financial support provided to families. Participants described that reducing the financial stress allowed families to focus on the information they were presented and the care of their child, “it helps them stay long enough to understand how the process is going to go because they know that they have this support” (nurse, Indus).

Additional quotations describing structural communication determinants are included in Table 2.

**TABLE 2:**
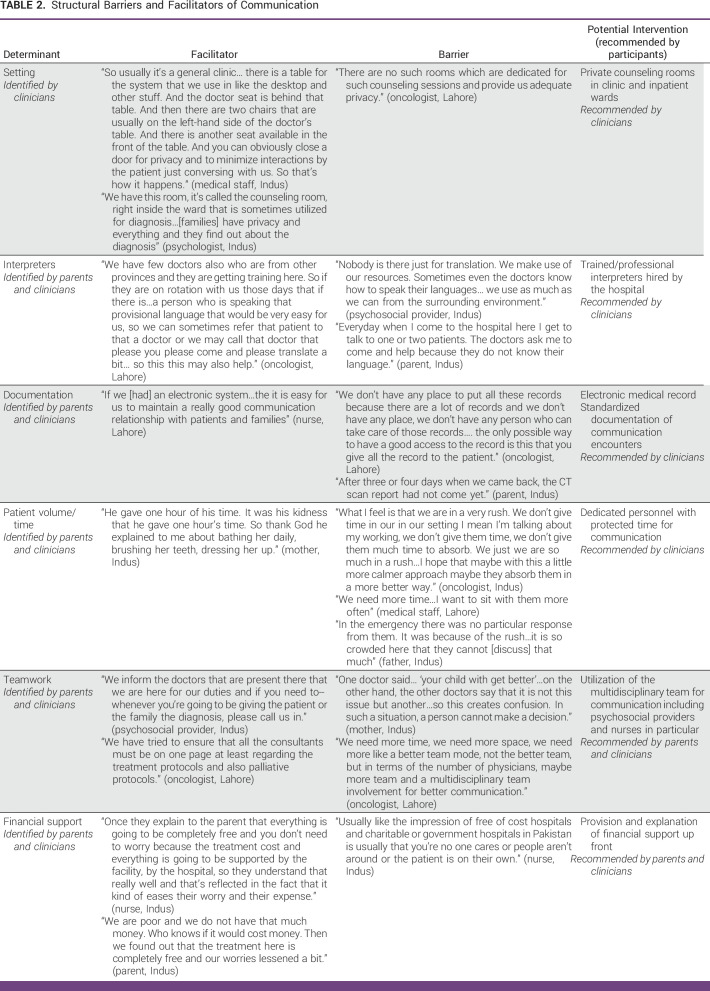
Structural Barriers and Facilitators of Communication

### Clinician-Level Barriers and Facilitators of Communication

Clinician factors that were perceived to affect quality communication included communication training, clinician distress, and the ability to have recurrent conversations.

None of the clinicians interviewed had received formal communication training. One clinician at Children's Hospital Lahore described this gap saying, “there are many others like me, who actually need the guidance…need to know how to reveal it to the patient and then how to communicate it to them.”

Clinicians further described ways in which they were personally affected by communication, one saying “It's hard to tell a parent about the diagnosis of cancer” (medical staff, Indus). At times, distress was evident to caregivers and affected caregiver reactions: “he started to seem worried; we got stressed” (mother, Indus). Clinician distress might have been exacerbated by limited professional boundaries as providers felt responsible for their patients day and night, communicating with them frequently over the phone.

Another factor raised by clinicians, although not identified by caregivers, was the ability to have recurrent conversations. Clinicians described the importance of repeating important information and the process of re-education or re-explanation.

Table 3 provides additional perspectives regarding clinician-level barriers and facilitators of communication.

**TABLE 3:**
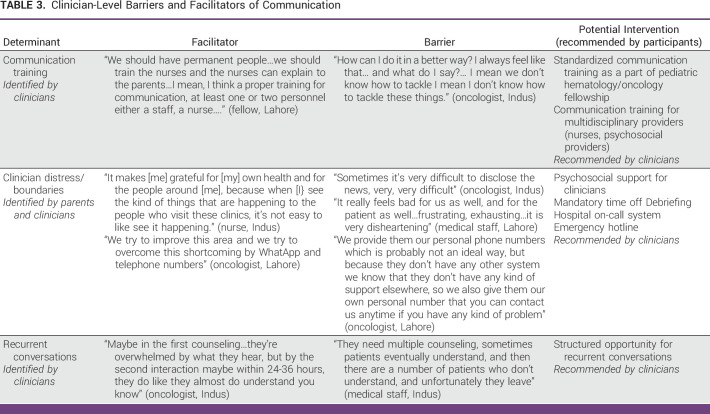
Clinician-Level Barriers and Facilitators of Communication

### Family-Level Barriers or Facilitators of Communication

Patient and family characteristics that affected communication with the medical team included demographic factors such as education or literacy, income status, primary language, and geography, as well as the child's diagnosis and relational factors including empowerment, social support, and split decision makers.

For the most part, clinicians described an easier time communicating with families that had higher levels of education. However, some clinicians disagreed regarding a link between education and communication: “I've seen many patients who have never been to school…but they can actually understand and follow the instructions… then there are parents who might have some education but still they fail to understand about or they miss appointments” (medical staff, Indus). Caregivers endorsed struggling with medical information because of limited formal education, and some described how educated family members helped them navigate the health care system.

Caregivers also discussed how limited financial resources affected every aspect of their child's care, including communication. One mother worried, “the advice they gave of giving her the best food, that is not something I can afford every day…I cannot do all that for [my child] which is supposed to be done” (mother, Indus). Caregivers and clinicians also noted that financial worries caused distress, which affected parents' ability to understand and absorb information.

Many families traveled long distances to the cancer center and spoke languages that their health care teams did not. Caregivers described how they overcame geography, and how the distance affected communication and decision making. Clinicians further emphasized how difficult it was to communicate with families from remote parts of Pakistan or neighboring countries, many of whom did not speak Urdu.

In addition, the type or stage of the patient's disease affected communication. Clinicians agreed that it was difficult to communicate in cases of relapse, or advanced disease, and easier to communicate with families of children who had leukemia than those with solid tumors.

Empowerment was often perceived by clinicians to facilitate communication as empowered families were more likely to engage and ask questions. Occasionally, however, clinicians portrayed family empowerment as a barrier to communication, leading parents to seek different advice from different members of the medical team.

Both clinicians and caregivers described how support from extended family, the medical team, or other parents facilitated communication. Unfortunately, geographic and financial constraints contributed to families being stripped of their usual support systems and at times led to situations in which the family decision makers were not all at the bedside, hindering communication.

Quotations describing factors affecting communication at the level of the family are included in Table 4.

**TABLE 4:**
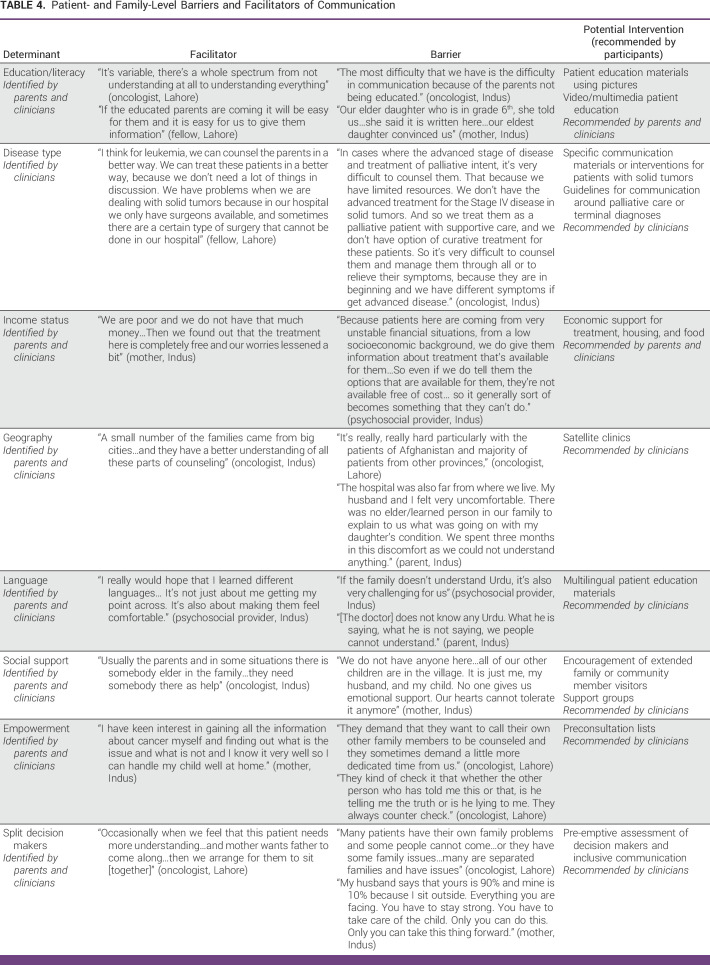
Patient- and Family-Level Barriers and Facilitators of Communication

### Interventions

Caregivers and clinicians identified current strategies and potential future interventions to improve communication within their settings. Photographs and videos of other children who had survived cancer gave caregivers hope and helped them accept treatment: “The pictures they showed of the children who were little and they have grown up now, I found hope, and how they got properly treated and now they have aged up” (mother, Indus). Caregivers also addressed the importance of providing housing and care free of cost, which decreased distress and allowed them to focus on medical information. Finally, caregivers noted that clinical team members, especially psychosocial providers and child life specialists, were sources of support.

Clinicians also described pictorial representations used for educational purposes and the financial support provided by institutions as ongoing interventions that facilitated communication. Some clinicians described general education sessions held for multiple families simultaneously or how they rearranged clinic schedules to give newer patients more time for discussion. Regarding future interventions, clinicians thought additional patient education materials, including booklets and videos, could be used to facilitate communication, particularly for families with language barriers: “we're trying to come up with a way or solution to make videos more accessible…or make it free of language” (psychologist, Indus). Clinicians at both hospitals also described how communication training for multidisciplinary providers would improve interactions with families. Finally, almost all clinicians thought about ways they could increase time with patients. This was described as an ongoing challenge and one of the reasons many clinicians gave out personal phone numbers. Some clinicians advocated for more physical space, but for most, the limitation was availability of clinical staff. At Indus, the psychosocial team provided additional support and recurrent conversations; clinicians at Children's Hospital Lahore wished for a similar model and described a communication team: “we should have a separate team for this and for the communication…we should have a separate counseling team” (oncologist, Lahore).

**FIG 1:**
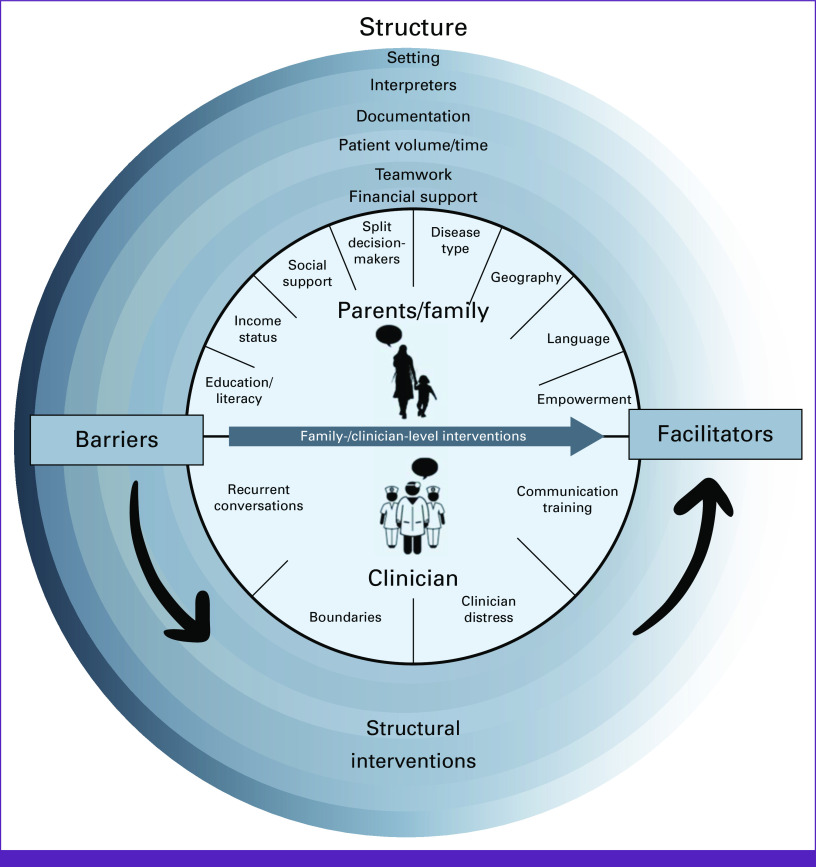
Multilevel factors affecting family-centered communication including interventions aimed at transforming barriers into facilitators.

Interventions described by clinicians and caregivers are mapped to each factor affecting communication in Tables 2-4. Figure 1 further illustrates how interventions at each level can transform communication barriers into facilitators.

## DISCUSSION

In this study, caregiver and clinician participants identified multilevel factors that facilitate or hinder family-centered communication in Pakistan. Many of these factors correlate with those previously identified in predominately high-income settings at the level of the individual (patient or clinician) and structural factors related to the hospital or care center.^16^ Each determinant provides an opportunity for interventional work that may improve communication in Pakistan and potentially other LMICs.

Although the two institutions included in this study are located over 600 miles apart and serve diverse patient populations, our findings demonstrated similarities regarding communication, including strengths and limitations. For example, although funding structures differ, both hospitals provide care at no cost to the family, allowing families to focus on their children. The financial burden of cancer care has been previously demonstrated to contribute to abandonment in LMICs.^17^ Our findings further support policy efforts aimed at improved coverage for care of children with cancer, including a National Cancer Control Plan in Pakistan,^18^ and emphasize the importance of communicating this coverage to patients and families.

In addition to financial resources, availability of clinical staff emerged as a significant limitation to high-quality communication. There are <50 trained pediatric oncologists in Pakistan. With an estimated 4,150 diagnoses each year, this is a ratio of 1:83 oncologists:patients, well above the recommended ratio of 1:50 in LMICs^19^ and five times the 1:15 we expect in high-income countries.^20^ Participants noted the time necessary for high-quality cancer communication and how difficult it was to find this time. Clinicians describe using WhatsApp to make themselves available at all times of day. While this is potentially effective in the short term, it was described as contributing to clinical distress and burnout, factors which have been related to clinician attrition in other LMICs,^21^ further driving limitations to human capacity.

Clinicians and caregivers emphasized the potential for interdisciplinary teamwork to reduce the communication burden. At Indus, a robust psychosocial team supported family-centered communication; this team was lacking at Children's Hospital Lahore. Nurses were important communicators at both institutions, but neither hospital had trained interpreters, and participants noted the detrimental effect this had on patient care. These findings demonstrate the importance of sustainable capacity building and educating an interdisciplinary workforce. In addition, although communication is recognized as a teachable skill,^2^ none of the clinicians included in our study received communication training.

The factors affecting communication at the level of the patient and family demonstrate how the diverse population in Pakistan may contribute to communication challenges. Certain populations, including those with lower income and education, rarer diagnoses, language or geographic barriers, and decreased social support and empowerment, may be at higher risk for poor communication. Communication interventions, including accessible patient education materials, support groups, and additional conversations targeted toward disadvantaged families, may be beneficial. In addition, these findings demonstrate how socioeconomic and cultural factors affect interactions between families and health care teams, demonstrating the need for culturally aware family-centered communication and individually tailored interactions.

This study explored pediatric cancer communication in Pakistan, a setting in which it has not previously been explored. The qualitative nature of the study allowed for in-depth investigation into themes that served as barriers and facilitators for family-centered communication at three distinct levels. However, results should be interpreted in the context of limitations. This study was conducted at only two of the nine centers that treat children with cancer in Pakistan, and all caregiver interviews were conducted at one center. While they are similar to themes identified in other settings and we are hopeful that the findings will resonate at all Pakistani cancer centers and lead to interventions at a national level, further work is necessary to determine the applicability of our results in other settings. Study materials were developed in English, and data analysis was conducted in English. Although our multilingual team reviewed data collection materials and thematic analysis throughout the study to decrease the impact of translation, our ability to interpret nuanced or linguistically dependent themes might have been limited. To recruit participants, we used purposive sampling. While we aimed to include a broad range of caregivers and multidisciplinary clinicians, our sample included a large number of physicians and it is possible that certain perspectives were excluded. Specifically, we did not include pediatric participants.

In conclusion, our findings illustrate the multifactorial contributors to patient- and family-centered communication. Future work should focus on applying these themes to diverse settings, and testing suggested interventions. While some interventional work may require diverse stakeholder engagement and financial resources, other efforts could be immediately explored by motivated local teams. Our hope is that these findings inspire a focus on improving communication not only at these two institutions but throughout Pakistan. Furthermore, we believe that many of the insights included here may be applicable to other LMICs and all settings providing care for diverse pediatric populations.

## Data Availability

Deidentified individual participant data will be made available, in addition to study protocols, the analysis plan, and the informed consent form. The data will be made available upon publication to researchers who provide a methodologically sound proposal for use in achieving the goals of the approved proposal. Proposals should be submitted to dylan.graetz@stjude.org.
